# Downregulation of VEGFR3 signaling alters cardiac lymphatic vessel organization and leads to a higher mortality after acute myocardial infarction

**DOI:** 10.1038/s41598-018-34770-4

**Published:** 2018-11-12

**Authors:** Taina Vuorio, Elias Ylä-Herttuala, Johanna P. Laakkonen, Svetlana Laidinen, Timo Liimatainen, Seppo Ylä-Herttuala

**Affiliations:** 1 A.I. Virtanen Institute for Molecular Sciences, University of Eastern Finland, P.O. Box 1627, FI-70211 Kuopio, Finland; 2 Research Unit of Medical Imaging, Physics and Technology, University of Oulu, Oulu, Finland; 3 Heart Center and Gene Therapy Unit, Kuopio University Hospital, P.O. Box 1777, FI-70211 Kuopio, Finland; 4 Department of Diagnostic Radiology, University Hospital of Oulu, P.O. Box 50, FI-90029 OYS Oulu, Finland

## Abstract

Heart has a wide lymphatic network but the importance of cardiac lymphatic system in heart diseases has remained unclear. Vascular Endothelial Growth Factor Receptor 3 (VEGFR3) is a key molecule in the development and maintenance of cardiac lymphatic vessels. Here we characterized the role of VEGFR3 in healthy hearts and after myocardial infarction (MI) by using sVEGFR3 transgenic mice expressing a soluble decoy VEGFR3 under K14 promoter and Chy mice which have an inactivating mutation in the VEGFR3 gene. Cardiac lymphatic vessels were significantly dilated in the healthy hearts of sVEGFR3 mice when compared to controls. Lymphatic vessels formed large sheet-like structures in Chy mice. Attenuated VEGFR3 signaling led to a more severe MI predisposing to a significantly higher mortality in sVEGFR3 mice than in control mice. sVEGFR3 mice displayed intramyocardial hemorrhages in the infarcted area indicating hyperpermeability of the vasculature. Furthermore, novel MRI methods TRAFF2 and TRAFF4 and histological analysis revealed a modified structure of the fibrotic infarcted area in sVEGFR3 mice. In conclusion, the downregulation of VEGFR3 signaling modifies the structure of cardiac lymphatic network and causes vascular leakiness and increased mortality after MI.

## Introduction

Lymphatic vessels were long considered as passive drainage conduits of extracellular fluid but their role has been extended since the mechanisms of their development and function in several pathophysiological processes have been identified^1^. Lymphatic system regulates many processes involved in cardiac physiology and pathology, such as inflammatory reactions^2^, tissue fluid balance^3^, reverse cholesterol transport^4^ and atherosclerosis^5,6^ which can eventually change heart function. Therefore, the role of lymphatic vessels in myocardial infarction (MI) and other heart conditions can be more significant than previously anticipated^7,8^.

The development of mouse cardiac lymphatic vessels starts at E12-14 when lymphatic endothelial cells (LECs) derived mainly from the common cardinal vein transmigrate to the dorsal and ventral surfaces of the heart and start to form lymphatic tubules and subsequently lymphatic capillary plexus^9,10^. In adults, capillary size lymphatic vessels cover the myocardium and subendocardium and also the atrioventricular and semilunar valves in most mammalian species^11,12^. It has been shown that cardiac lymph flow begins from small endocardial lymphatics and continues through myocardium into subepicardial capillaries that converge into larger collecting lymphatic vessels. Finally, cardiac lymph passes through the mediastinal lymph nodes into the thoracic duct^13^.

Only a few studies have focused on the function of lymphatic vessels in regulating cardiac physiology or their role in cardiac pathologies. The effect of cardiac lymph flow impairment has been studied by blocking ventricular and mediastinal lymphatic ducts in large animals (reviewed by Cui^14^). In these studies, the obstruction of lymphatic flow led to subepicardial edema, depressed left ventricle (LV) contractile function and hemorrhages. Cardiac arrhythmias have also been associated with lymphedema both in humans and in animal models^14^. On the other hand, lymphangiogensis has been observed in rats^15^ and humans^11^ after MI. In addition, lymphangiogenic therapy with vascular endothelial growth factor receptor 3 (VEGFR3)-specific vascular endothelial growth factor C (VEGF-C) protein improved LV function after MI in mice^9^ and resolved cardiac edema and fibrosis in rats^16^. It has also recently been shown in a human phase 1 clinical trial that the gene transfer of another VEGFR3 ligand, VEGF-D, improves cardiac blood flow in refractory angina patients^17^.

Here we analyzed the role of VEGFR3 in cardiac lymphatic vessel morphology and cardiac function in healthy hearts and after MI in mice. We used two mouse models with defective VEGFR3 signaling: sVEGFR3 mice expressing soluble decoy VEGFR3 (sVEGFR3) and Chy mice with inactivating point mutation in VEGFR3 gene (Chy)^6^. The blocking of the VEGFR3 signaling altered the structure of cardiac lymphatics in healthy hearts but did not affect cardiac function. After MI, sVEGFR3 mice had significantly higher mortality than the control littermates, intramyocardial hemorrhages, a reduced capability to respond to lymphangiogenic signals and a modified structure of the infarcted area.

## Results

### sVEGFR3 mice have higher mortality after MI than control mice

**Figure 1:**
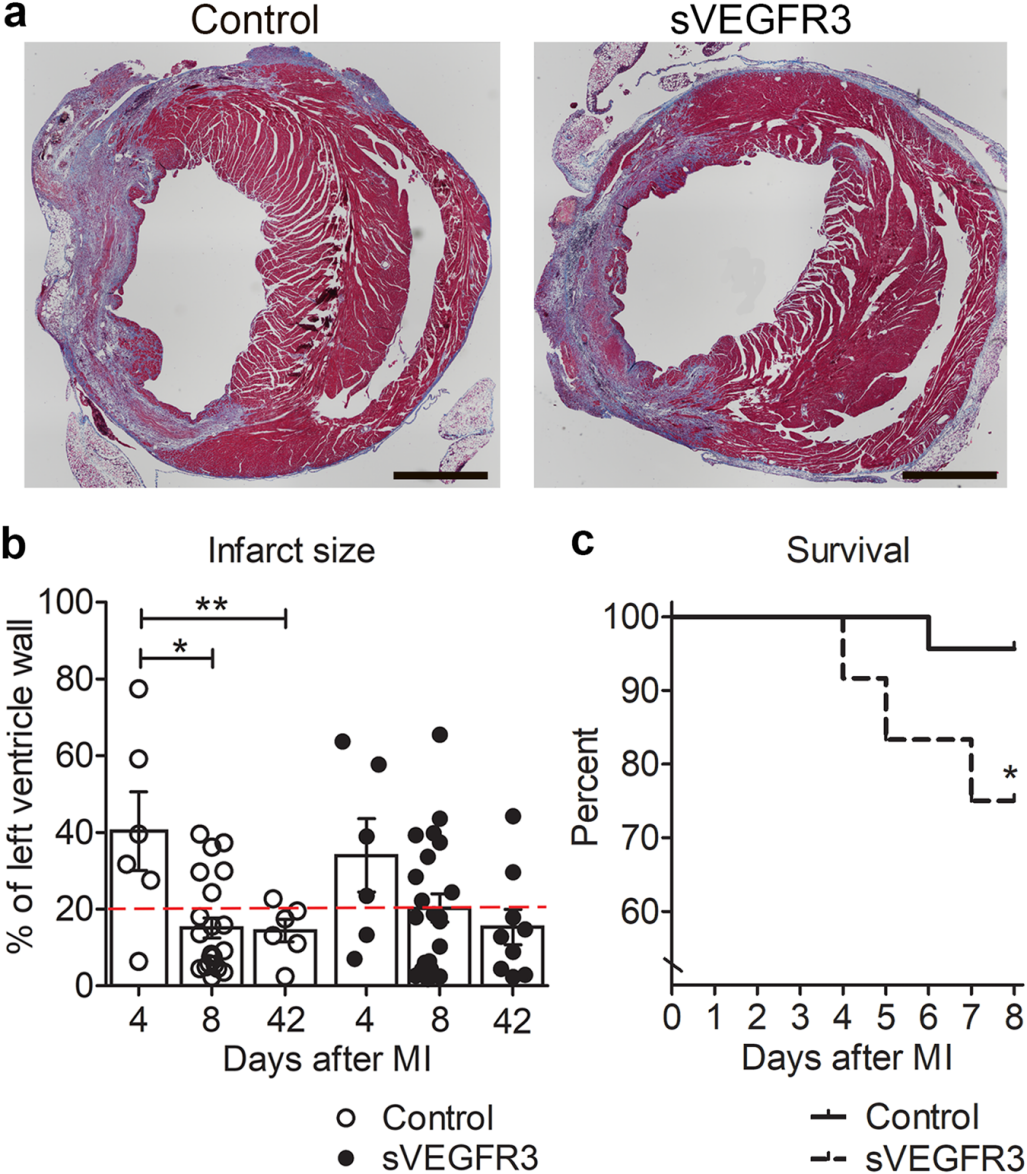
MI leads to higher mortality in sVEGFR3 mice compared to the controls. (**a**) Representative images of heart cross-sections stained with Masson’s Trichrome staining show collagen accumulation and necrotic infarction scar in sVEGFR3 and control mice 8 days post-MI. (**b**) Quantification of infarction area sizes 4, 8 and 42 days post-MI (n = 6/group, n = 22/group and n = 6–9, respectively). Infarct areas above the red dashed line (20%) are considered large. (**c**) Mortality of sVEGFR3 and control mice during 8 day follow-up after LAD ligation (n = 23–24/group). Scale bar in (**a**) is 1,000 µm. Values represent mean ± SEM. Statistical analyses were performed using two-way ANOVA with Bonferroni’s post-hoc test or Kaplan-Mayer with log-rank test for survival curve. ^*^P < 0.05, ^**^P < 0.01.

To study the role of lymphatic vessels in MI, the anterior branch of the left descending coronary artery (LAD) was ligated in sVEGFR3 and control mice to generate an anteroapical infarctionin the LV wall (LVW). In order to analyze different stages of myocardial healing after LAD ligation, mice were followed for 4, 8 or 42 days. LAD ligation induced typical signs of MI, such as inflammatory cell accumulation, fibrosis and scar formation (Fig. 1a). The largest infarction areas were detected already 4 days after MI both in sVEGFR3 mice and control mice. The average infarction area was decreased at the later time points in both groups reaching significance in control mice (39.6% at 4 days post-MI vs. 15.1% at 8 days post-MI, P < 0.01 and 14.4% 42 days post-MI, P < 0.01) (Fig. 1b). During the 8 day follow-up, the mortality of sVEGFR3 mice was significantly higher compared to the control group (25% vs. 4%, respectively, P < 0.05) (Fig. 1c). This might be explained by a higher proportion of the larger infarction areas in sVEGFR3 mice than in control mice: 40.9% of sVEGFR3 mice had infarction areas spanning more than 20% of the LVW, whereas only 27.2% of control mice had these large infarcted areas (Fig. 1b). Furthermore, 5 out of 6 sVEGFR3 mice that died during the 8 day follow-up had infarction areas larger than 20% of the LVW (Fig. 1b).

### MI induced changes in the cardiac function in sVEGFR3 mice and control mice

**Figure 2:**
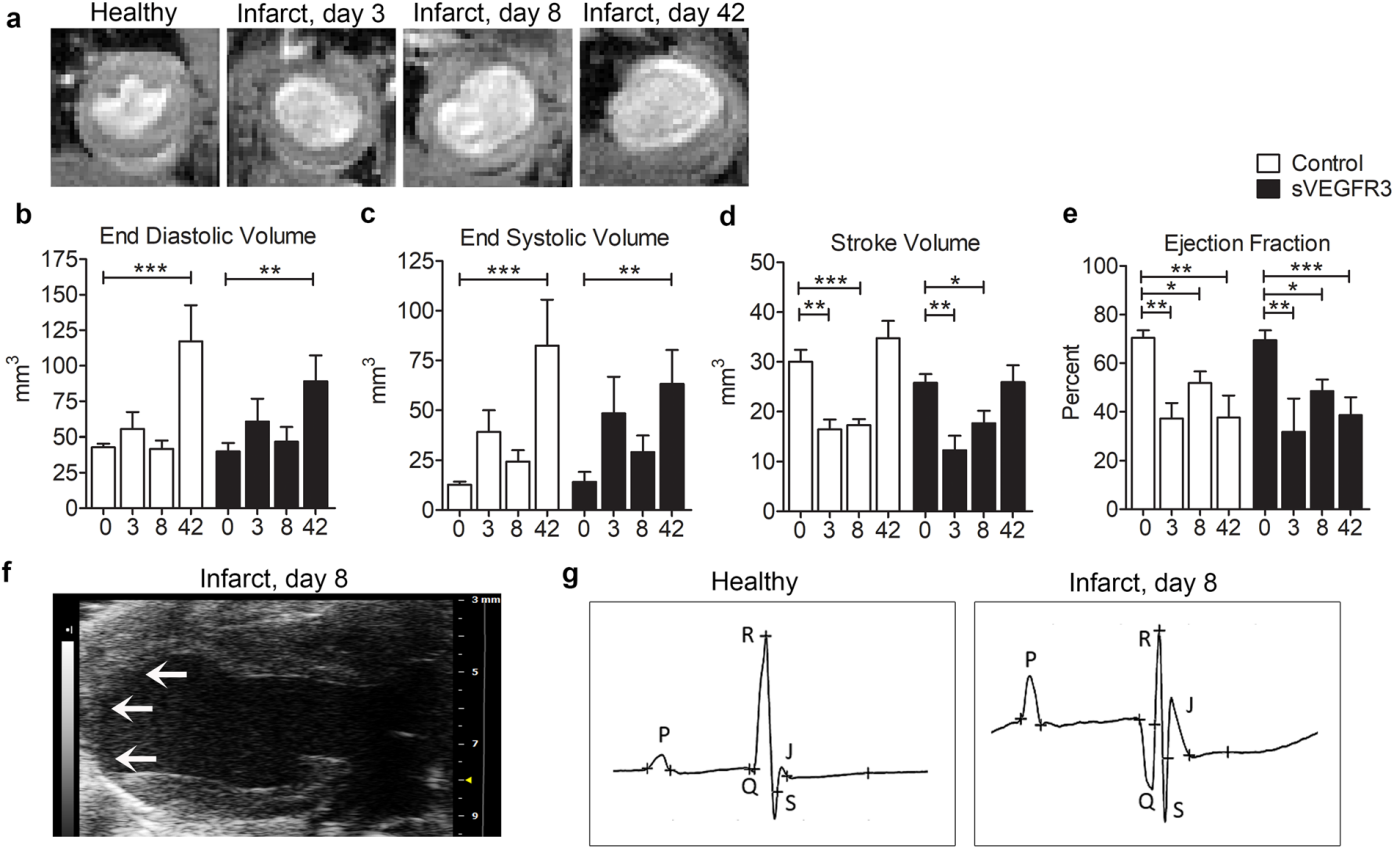
sVEGFR3 mice and control mice have similar heart function. **(a)** Examples of MRI cine images from healthy and infarcted hearts 3, 8 and 42 days post-MI show dilatation of the heart and thinning of the LVW. **(b**,**e)** EDV (**b**), ESV (**c**), SV (**d**) and EF (**e**) measured from cine MRI images in healthy hearts (0) and 3, 8 and 42 days post-MI (n = 8–11/group, n = 4/group, n = 14–18/group and n = 6–9/group, respectively). (**f**) An example of echocardiogram image from infarcted heart shows clear thinning of the apical LVW. Infarction scar is shown by arrows. (**g**) Examples of ECG profiles show pathological Q waves and changes in R and S wave amplitudes in an infarcted heart compared to a healthy heart. Values represent mean ± SEM. Statistical analyses were performed using two-way ANOVA with Bonferroni’s post-hoc test. ^*^P < 0.05, ^**^P < 0.01, ^***^P < 0.001.

Cardiac magnetic resonance imaging (cMRI) was utilized to measure heart function and to visualize the infarction area. The thinning of the LVW and the dilatation of the LV were easily detectable from cine MRI images at all time points after MI (Fig. 2a). LV volumes in diastole (EDV) and systole (ESV) were measured from cine MRI images (Fig. 2b,c). Additionally, stroke volume (SV) and ejection fraction (EF) were calculated by using EDV and ESV values (Fig. 2d,e). Compared to healthy hearts, ESV values increased slightly already 3 and 8 days after MI (Fig. 2c) and EDV and ESV values were significantly increased 42 days post-MI both in sVEGFR3 mice (EDV: 40.0 mm^3^ in healthy hearts vs. 89.1 mm^3^ 8 days post-MI, P < 0.01) and in control mice (EDV: 42.8 mm^3^ in healthy hearts vs. 117.2 mm^3^ at 42 days post-MI, P < 0.001), which confirms the dilatation of the LVW (Fig. 2b,c). Increased ESV values caused SV values to decrease significantly 3 and 8 days post-MI both in sVEGFR3 mice (25.8 mm^3^ in healthy hearts vs. 12.3 mm^3^ at 3 days post-MI, P < 0.01 and 17.6 mm^3^ at 8 days post-MI, P < 0.05) and in control mice (30.0 mm^3^ at in healthy hearts vs. 16.4 mm^3^ at 3 days post-MI, P < 0.01 and 17.3 mm^3^ at 8 days post-MI, P < 0.05) (Fig. 2d). Compared to healthy hearts, EF was significantly decreased 3, 8 and 42 days post-MI sVEGFR3 mice (69.4% vs. 31.7%, P < 0.01, 48.4%, P < 0.05 and 38.6%, P < 0.001, respectively) and in control mice (70.4% vs. 37.2%, P < 0.01, 51.8%, P < 0.05 and 37.6%, P < 0.01, respectively) indicating progressive reduction in the pumping efficacy of the infarcted hearts (Fig. 2e).

Echocardiography was performed for the healthy hearts and 7 and 35 days post-MI to obtain more knowledge about the changes in heart function caused by LAD ligation. The thinning of the LVW was easily visible from long-axis view (LAX) of echocardiography 8 days post-MI (Fig. 2f). In addition, electrocardiograms (ECG) were recorded during echocardiography. As previously described^18^, pathological Q waves appeared in ECG post-MI both in sVEGFR3 and control mice (Fig. 2g). In addition, clear changes in R wave and S wave amplitudes were detected. ECG profiles were similar between sVEGFR3 mice and controls both in healthy and post-MI states (Table 1).

**Table 1:** Quantification of ECG profiles in healthy hearts and 8 days and 35 days post-MI in sVEGFR3 mice and the control mice.

		QRS (ms)	Q dur (ms)	Amp Q (mV)	Amp R (mV)	Amp S (mV)	HR (bpm)
Healthy	Control	10.1	1.0	−0.2	12.5	−6.4	454
	sVEGFR3	10.4	0.4	0.0	11.8	−5.1	463
Infarcted, 7 days post-MI	Control	12.3	3.1	−0.8	9.3	−4.8	480
	sVEGFR3	12.2	2.7*	−0.7	8.1	−4.0	486
Infarcted, 35 days post-MI	Control	12.1	5.5***	−1.4	4.5**	−2.4**	507
	sVEGFR3	10.4	2.1^#^	−1.2	6.9*	−5.4	489

QRS: Duration of QRS complex, Q dur: Q wave duration, Amp Q: Q wave amplitude, Amp R: R wave amplitude, Amp S: S wave amplitude, HR: heart rate. bpm: beats per minute. n = 9–10/group in healthy hearts, n = 17–20/group 7 days post-MI and n = 5–9/group 35 days post-MI. Values represent mean ± SEM. Statistical analyses were performed using one-way ANOVA with Bonferroni’s post-hoc test. ^*^P < 0.05, ^**^P < 0.01, ^***^P < 0.001 when compared to healthy heart, ^#^P < 0.05 when compared to control mice at the same time point.

### Cardiac lymphatic vessel morphology and lymphangiogenesis are modified in sVEGFR3 mice

**Figure 3:**
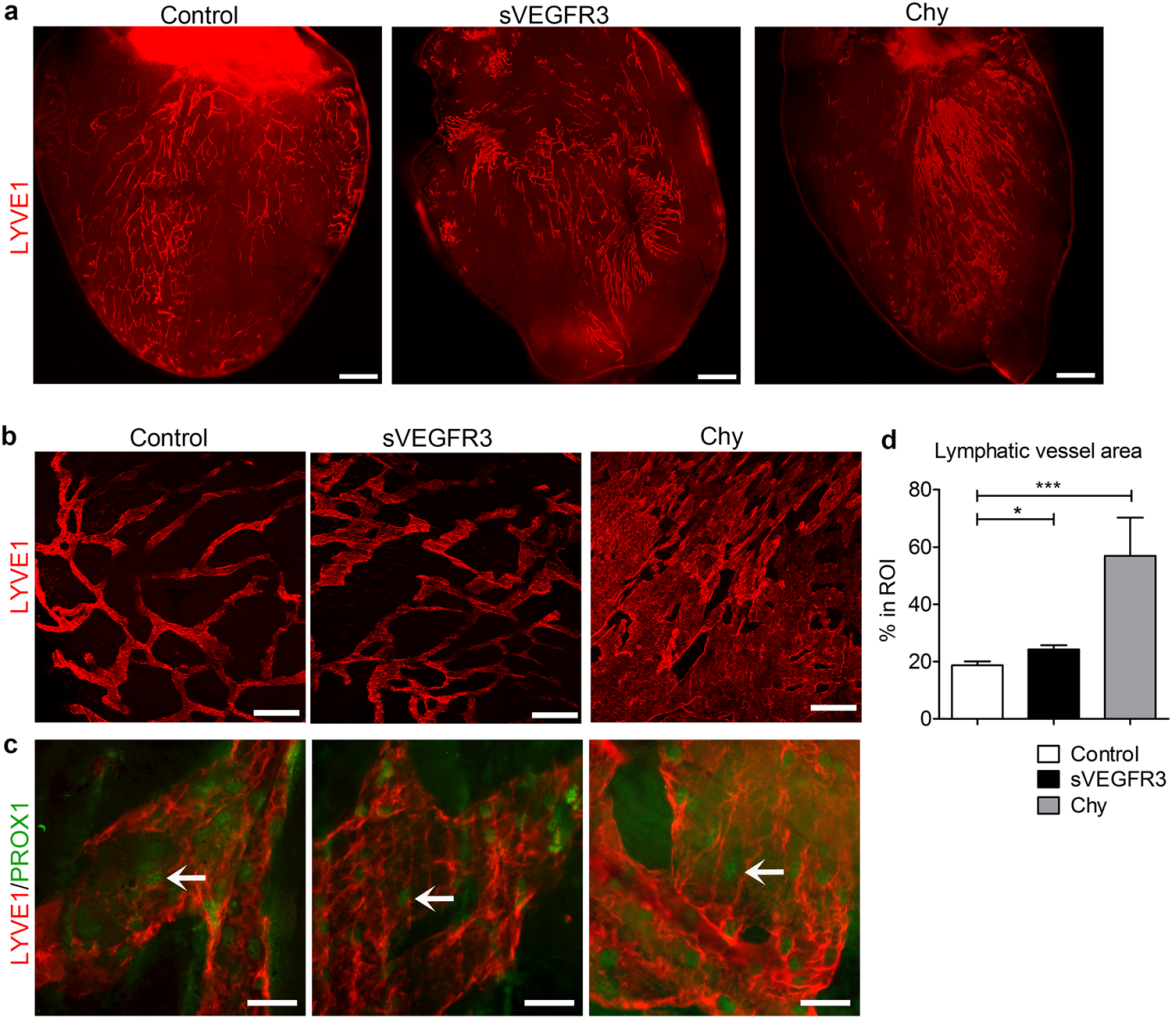
Cardiac lymphatic vessels are dilated in healthy sVEGFR3 mice and completely loose their organization in Chy mice. (**a**) Representative images of LYVE1 positive subepicardial cardiac lymphatics in the anterior side of the heart in the healthy control mice, sVEGFR3 mice and Chy mice. (**b**) Representative images of LYVE1 positive cardiac lymphatic vessels in LVW in the healthy control mice, sVEGFR3 mice and Chy mice. (**c**) Representative images of PROX1 and LYVE1 double-stainings in LVW in the healthy control mice, sVEGFR3 mice and Chy mice. Arrows indicate PROX1-positive LEC nuclei. (**d**) Quantification of LYVE1 stainings (n = 11/control mice, n = 17/sVEGFR3 mice and n = 4/Chy mice). Scale bar in (**a**) is 1,000 µm, in (**b**) 200 µm and in (**c**) 50 µm. Values represent mean ± SEM. Statistical analyses were performed using Student’s t-test. ^*^P < 0.05, ^***^P < 0.001.

To determine the effect of attenuated VEGFR3 signaling on lymphatic vessel morphology, 3D hierarchy of cardiac lymphatic vessels was studied after immunostaining with LYVE1 marker from the anterior surface of the heart (Fig. 3a) and specifically from the anterioapical pieces of the heart (Fig. 3b). Lymphatic vessels were shown to be dilated in otherwise healthy sVEGFR3 mice in comparison to control mice. In Chy mice, the vessels had completely lost their fishnet-like organization and formed large sheet-like structures (Fig. 3a,b). Nuclear PROX1 staining indicated that the abnormal morphology did not result from the proliferation of LECs (Fig. 3c). sVEGFR3 mice had significantly increased lymphatic vessel area (24.2%) compared to control mice (18.7%) (P < 0.05). In Chy mice, lymphatic area in the region-of-interest (ROI) varied from approximately 39% up to 90% (P < 0.001) (Fig. 3d). Gender or diet did not affect the cardiac lymphatic vessel organization (data not shown).

**Figure 4:**
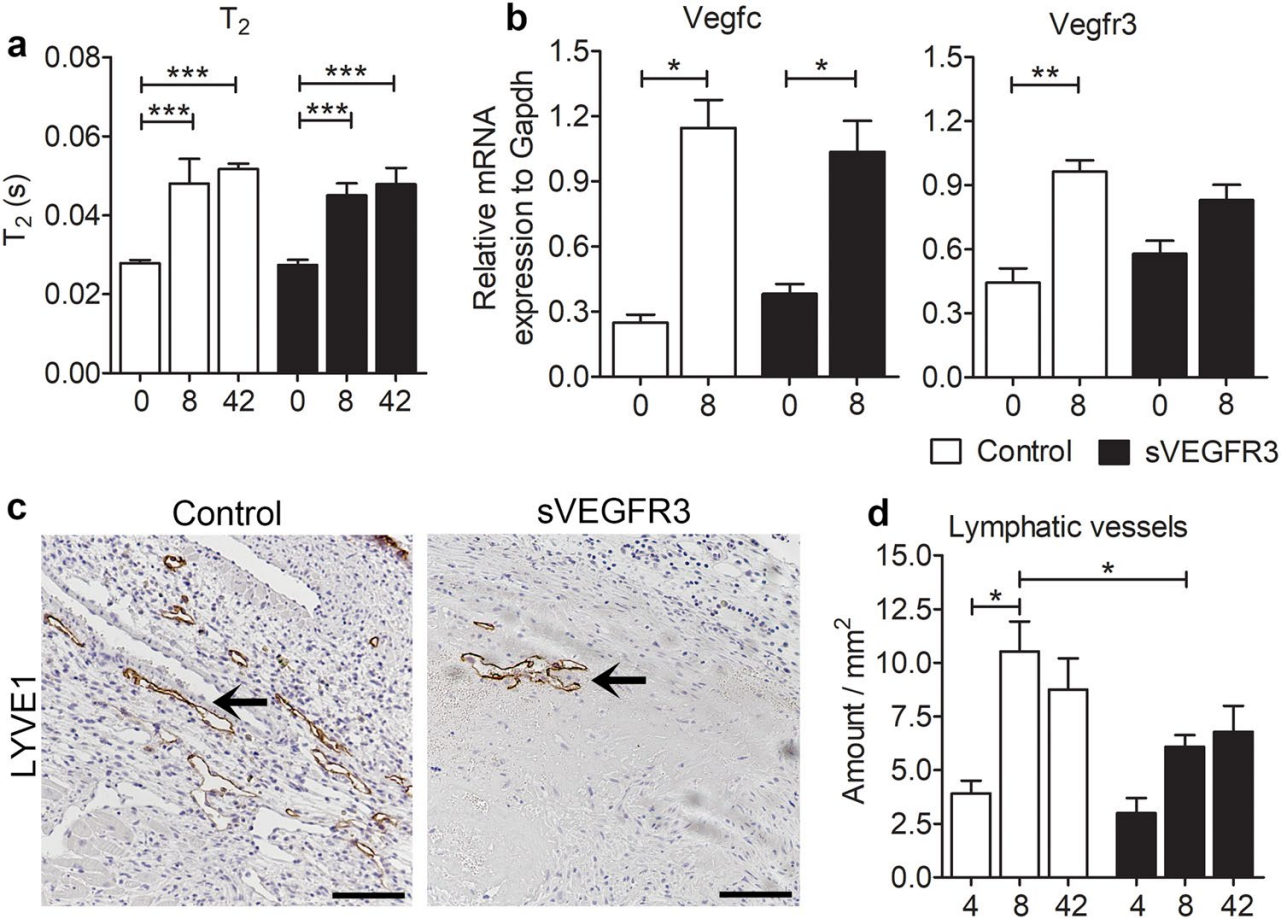
Cardiac lymphatic vessels in sVEGFR3 mice are unable to respond to lymphangiogenic signals after MI. (**a**) MRI T_2_ relaxation times in healthy hearts and 8 and 42 days post-MI (n = 8–9/group, n = 5–6/group and n = 8–9/group, respectively). (**b**) RT-qPCR analyses for Vegf-c and Vegfr3 mRNA in healthy hearts and 8 days post-MI (n = 2–3/group and n = 9–15/group, respectively). (**c**) Representative images of LYVE1 positive cardiac lymphatic vessels in heart cross-sections 8 days post-MI in sVEGFR3 and control mice. Arrows indicate LYVE-1 positive lymphatic vessels. (**d**) Quantification of LYVE1 stainings 4, 8 and 42 days post-MI (n = 5–6/group, n = 12–16/group and n = 6–9/group, respectively). Scale bar in (**c**) is 125 µm. Values represent mean ± SEM. Statistical analyses were performed using two-way ANOVA with Bonferroni’s post-hoc test or Student’s t-test. ^*^P < 0.05, ^**^P < 0.01, ^***^P < 0.001.

Attenuated lymphatic vessel function leads to the accumulation of tissue fluids^19^. Long-lasting edema can influence the development of fibrosis and thereby alter the healing after MI^20^. Previously, T_2_ weighted MRI method has been used to noninvasively visualize and quantitate edematous regions in humans and animal models^21–23^. Here we used T_2_ weighted MRI to measure cardiac edema in healthy hearts, 8 days and 42 days post-MI (Fig. 4a). T_2_ relaxation times were significantly increased in infarcted hearts at both time points compared to the healthy hearts (0.027 s at d0 vs. 0.046 s 8 days after MI, p < 0.001 and 0.047 s 42, p < 0.001 in sVEGFR3 mice).

To resolve the accumulation of fluids and inflammatory cells in the myocardium after MI, lymphangiogenesis is activated both in the infarcted regions and in healthy LVW^16^. Here we analyzed the expression of VEGFR3 and VEGF-C with RT-qPCR to determine if these main lymphangiogenic regulators are activated after MI. VEGF-C was upregulated both in sVEGFR3 and control mice when compared to healthy hearts 8 days after MI (P < 0.05) (Fig. 4b). Also, the expression of VEGFR3 was considerably increased in sVEGFR3 mice and it was significantly upregulated in control mice (P < 0.01). Furthermore, the localization and number of lymphatic vessels after MI was evaluated from the cross-sections of LVW stained with LYVE1 antibody (Fig. 4c,d). In the controls, lymphatic vessels formed a dense network indicating activated lymphangiogenesis especially in the border zone of the infarcted area whereas in sVEGFR3 mice lymphatic vessels were nearly absent 8 days after MI (Fig. 4c). The amount of lymphatic vessels was similar in sVEGFR3 mice and control mice 4 days after MI but it was significantly increased in control mice 8 days after MI (3,9 /mm^2^ 4 days post-MI vs. 10.5 /mm^2^ 8 days post-MI. P < 0.05) and remained at high level until the day 42 (Fig. 4d) confirming strongly activated lymphangiogenesis in control mice during the healing of the myocardium after MI. In contrast, the amount of lymphatic vessels increased only slightly in sVGEFR3 mice during the follow-up and the amount was significantly lower in sVEGFR3 mice compared to control mice 8 days after MI (6.1/mm^2^ vs. 10.5/mm^2^, respectively. P < 0.05) (Fig. 4d) indicating attenuated capability to respond to lymphangiogenic signals.

### sVEGFR3 mice display intramyocardial hemorrhages

**Figure 5:**
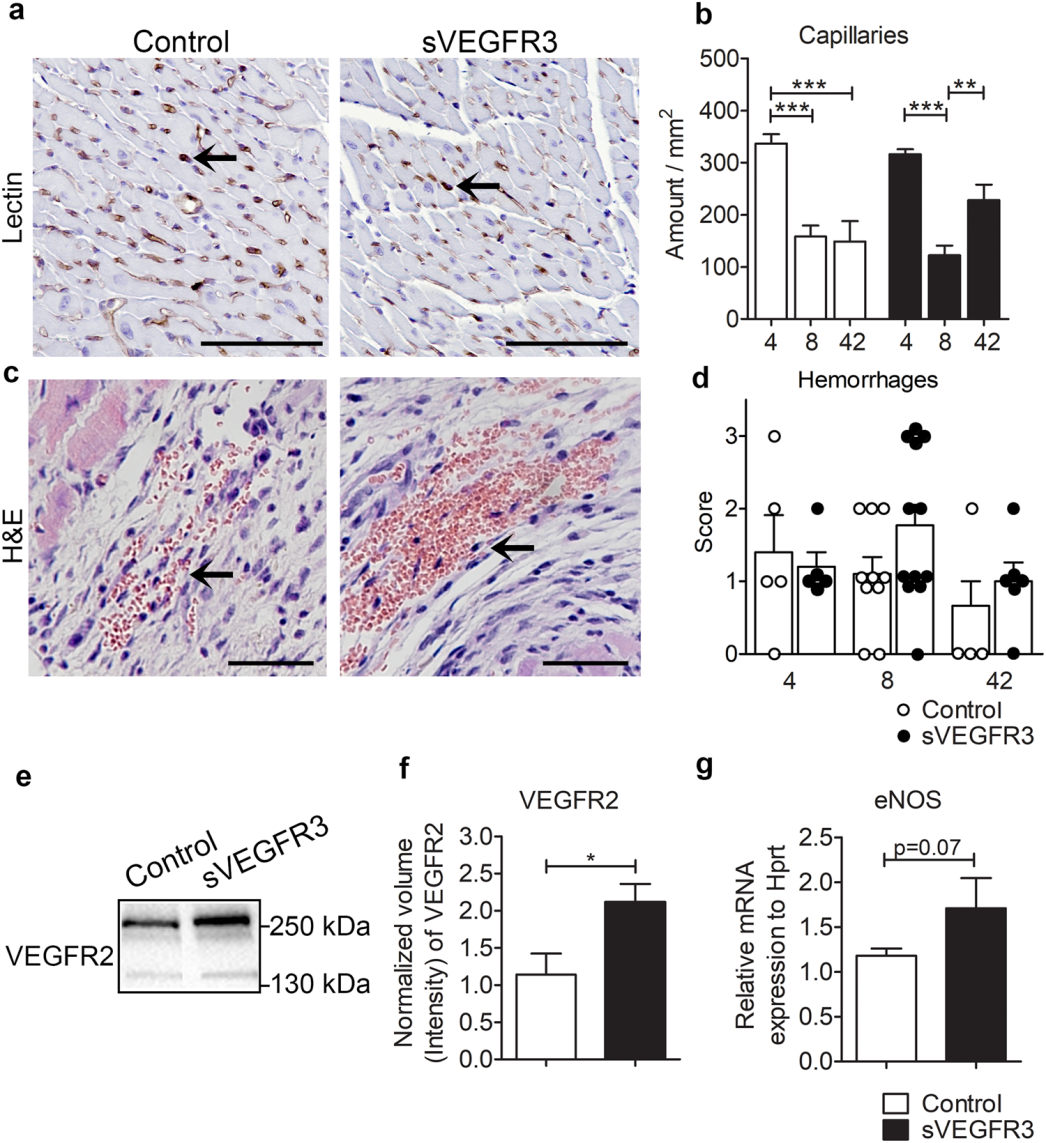
Histological examination revealed hemorrhages in the infarcted areas of sVEGFR3 mice. (**a**,**b**) Representative images of Lectin positive cardiac blood capillaries in heart cross-sections 8 days post-MI in sVEGFR3 and control mice. Arrows indicate Lectin positive capillaries. (**b**) Quantification of Lectin stainings 4, 8 and 42 days post-MI (n = 6/group, n = 21/group and n = 6–9/group). (**c**) Representative images of intramyocardial hemorrhages in control mice and sVEGFR3 mice. Arrows indicate red blood cells (RBCs) (**d**) Scoring of RBCs in infarcted area of LVW 4, 8 and 42 days post MI (n = 5/group, n = 10–12/group and n = 4–5/group, respectively). Score 0: no RBCs, 1: few RBCs, 2: hemorrhages 3: multiple hemorrhages. (**e**,**f**) Western blot (**e**) and quantification (**f**) of VEGFR2 in the healthy hearts of sVEGFR3 mice (n = 4–5/group). (**g**) qPCR analysis of eNOS in the hearts of sVEGFR3 mice and controls 8 days after the infarction (n = 9–15/group). Scale bar is (**a**) 100 µm and in (**c**) 50 µm. Values represent mean ± SEM. Statistical analyses were performed using two-way ANOVA with Bonferroni’s post-hoc test or Student’s t-test. ^*^P < 0.05, ^**^P < 0.01, ^***^P < 0.001.

To evaluate the angiogenic response after MI, blood capillaries were stained with Lectin (Fig. 5a) and quantified from the border zone of the infarcted area (Fig. 5b). The highest amount of capillaries was detected 4 days after MI in both groups and it was significantly decreased at the later time points. The amount of capillaries was equal between sVEGFR3 mice and control mice indicating similar responses to angiogenic signals. Unexpectedly, most of the sVEGFR3 mice that died during the 8 day follow-up displayed large hemorrhages in the infarcted area indicating leakiness from blood vessels (Fig. 5c). The scoring of the hemorrhagic areas of infarcted areas covering more than 10% of LVW revealed large accumulations of erythrocytes especially in sVEGFR3 mice followed for 8 days (Fig. 5d). As downregulation of VEGFR3 signaling has been shown to increase VEGFR2 levels and thereby vascular permeability^24^, we measured the levels of VEGFR2 in healthy hearts by Western blotting (Fig. 5e). The amount of VEGFR2 protein was significantly increased in the healthy hearts of sVEGFR3 mice (P < 0.05) (Fig. 5f). Additionally, the expression of endothelial nitric oxide synthase (eNOS) was upregulated in sVEGFR3 mice 8 days after MI compared to controls (p = 0.07) indicating that the expression of sVEGFR3 may induce changes in the heart vasculature during MI by modulating VEGFR2-mediated vascular permeability.

### sVEGFR3 mice and control mice had equal levels of inflammatory cells

**Figure 6:**
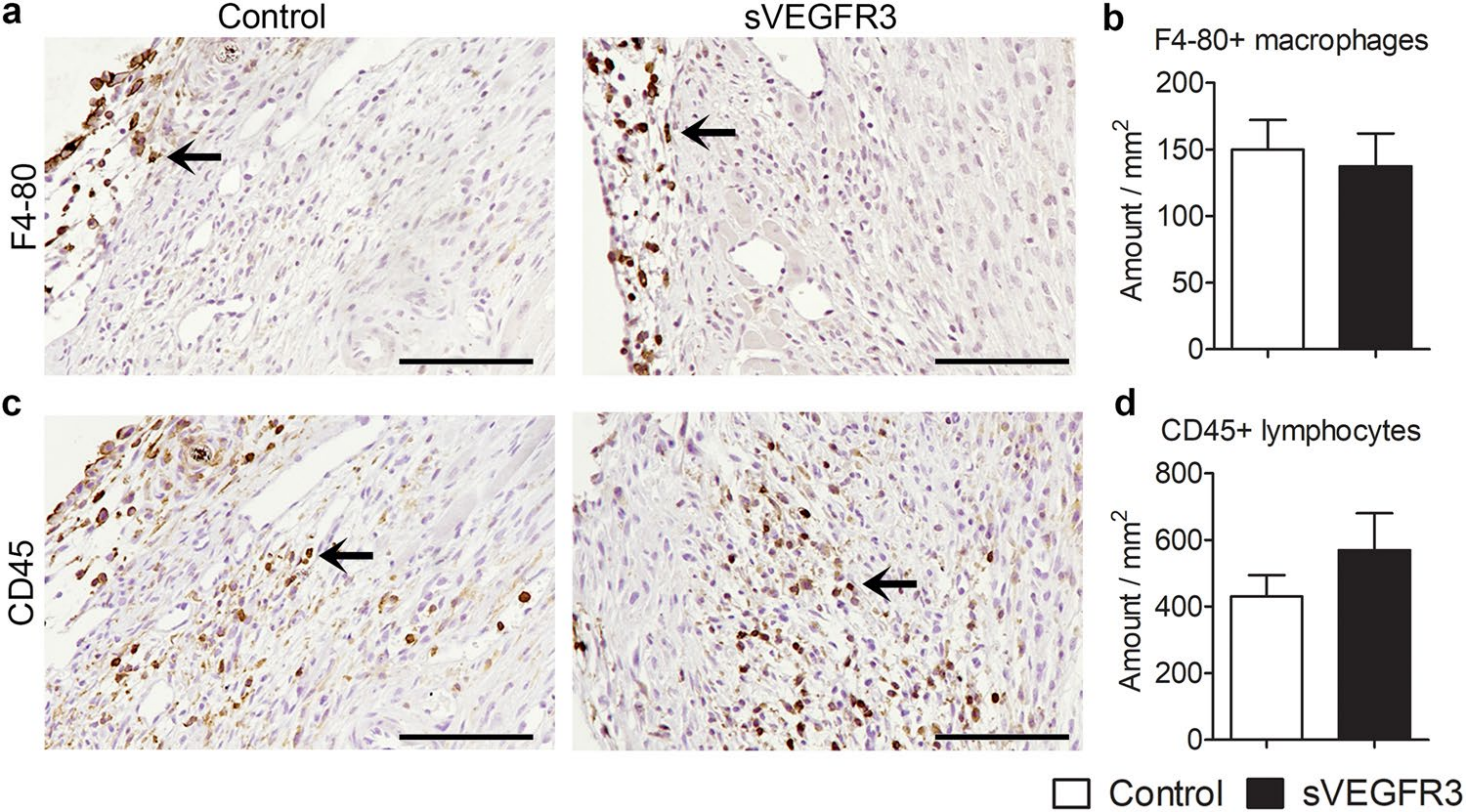
sVEGFR3 mice and control mice display similar amounts of inflammatory cells in LVW. (**a**,**b**) Representative images (a) and quantification (b) of F4–80 stainings 8 days post-MI (n = 14–17/group). (**c**,**d**) Representative images (c) and quantification (d) of CD45 stainings 8 days post-MI (n = 16–17/group). Scale bar in (**a**) and (**c**) is 100 µm. Values represent mean ± SEM. Statistical analyses were performed using Student’s t-test.

Myocardial necrosis after MI triggers the recruitment of inflammatory cells that clear the wound from dead cells and matrix debris. To analyze the inflammatory reaction after MI, macrophages and lymphocytes were stained with F4-80 and CD45 antibodies, respectively, from the cross-sections of infarcted hearts. Eight days after MI, F4-80+ macrophages were mainly located in the epicardium of LVW (Fig. 6a) whereas CD45+ lymphocytes were scattered throughout the infarction area (Fig. 6b). Healthy areas were almost completely devoid of inflammatory cells. Even though sVEGFR3 mice had a slight increase in the amount of lymphocytes, no significant changes in the amount of inflammatory cells in LVW were detected between control mice and sVEGFR3 mice 8 days after MI (Fig. 6c).

### Histology and novel MRI methods revealed changes in the structure of infarcted areas in sVEGFR3 mice

**Figure 7:**
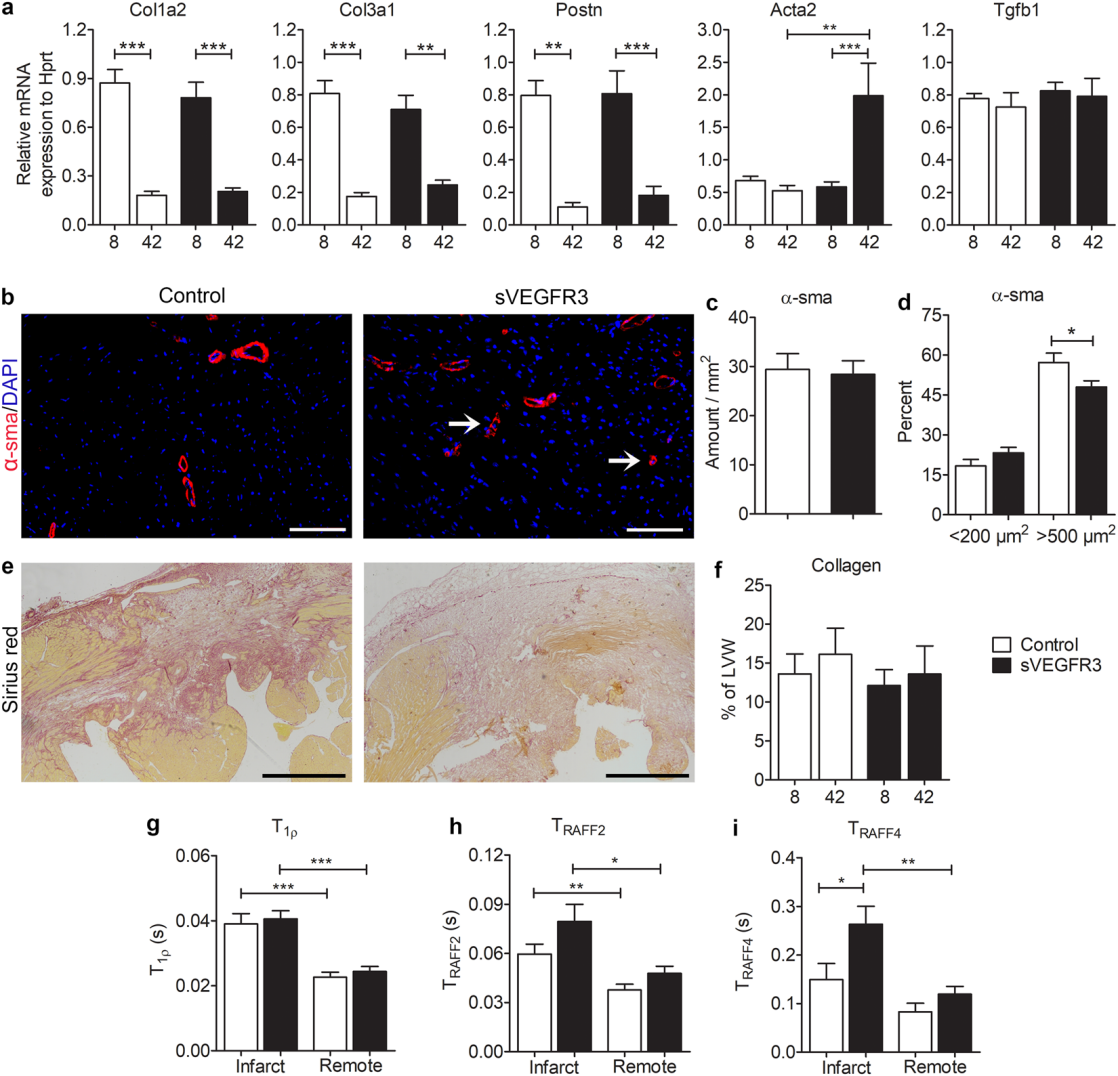
Histology and novel MRI methods display differential fibrosis in sVEGFR3 mice when compared to the controls. (**a**) RT-qPCR analyses for Col1a2, Col3a1, Postn, Acta2 and Tgfb1 mRNAs 8 and 42 days post-MI (n = 14–18/group and n = 4–9/group, respectively). (**b**) Representative images of α-sma staining in heart cross-sections 42 days post-MI in sVEGFR3 and control mice. Arrows point at individual α-sma positive cells. (**c**) Amount of α-sma positive area in the healthy LVW in sVEGFR3 and control mice (n = 6–9/group). (**d**) Proportion of small (<200 µm) and large (>500 µm) α-sma positive structures in the healthy LVW (n = 6–9/group). (**e**) Representative images of Sirius Red staining in heart cross-sections 8 days post-MI in sVEGFR3 and control mice. (**f**) Quantification of collagen in LVW from Sirius Red stainings 8 and 42 days post-MI (n = 17–18/group and n = 6–9/group, respevctively). (**g**,**i)** MRI T_1ρ_ (**g**), T_RAFF2_ (**h**) and T_RAFF4_ (**i**) relaxation times 7 days post-MI in infarcted and remote regions (n = 5–11/group). Scale bar in (**e**) is 100 µm and in (**h**) 500 µm. Values represent mean ± SEM. Statistical analyses were performed using two-way ANOVA with Bonferroni’s post-hoc test or Student’s t-test. ^*^P < 0.05, ^**^P < 0.01, ^***^P < 0.001.

After the infiltration of inflammatory cells, proinflammatory signaling is suppressed in the infarcted areas and fibroblasts turn into activated myofibroblasts. Myofibroblasts produce large amounts of collagens that provide tensile strength for the myocardial wall and protect it from rupture^25^. To assess the production of fibrotic and contractile proteins in the myocardium after MI, RT-qPCR analyses were performed for collagens (Col1A2 and Col3A1), periostin (Postn), smooth muscle cell actin (Acta2) and transforming growth factor beta 1 (Tgfb1) (Fig. 7a). The expression of collagens and Postn were significantly decreased 42 days after MI compared to the expression in the 8 day samples. The expression of both Col1A2 and Col2A3 were slightly but not significantly decreased in sVEGFR3 mice compared to the controls 8 days post-MI. Acta1 was strongly upregulated in sVEGFR3 mice 42 days after MI ligation indicating accumulation of α-SMA positive cells (primarily myofibroblasts) in the LVW. This finding was supported by immunohistochemical staining for α-sma which revealed the accumulation of individual α-sma positive cells and small arterioles in the healthy LVW of sVEGFR3 mice 42 days after MI whereas α-sma was more prominently expressed in the larger arteries in control mice (Fig. 7b). sVEGFR3 mice and control mice had similar amounts of α-sma positive structures (Fig. 7c) but sVEGFR3 mice displayed a trend towards smaller α-sma positive cells and arterioles whereas the proportion of large α-sma positive arteries was significantly higher in control mice than in sVEGFR3 mice (57.1% vs. 48.0%, P < 0.05, respectively)(Fig. 7d).

The amount of fibrosis was further analyzed from heart cross-sections stained with Picro-Sirius red that allowed the visualization of collagen types I and III. Interestingly, the intensity of collagen staining was fainter in several sVEGFR3 mice compared to control mice 8 days post-MI (Fig. 7d), which indicates a different composition of the fibrotic area. However, there were no differences in the total amount of collagen in LVW between sVEGFR3 mice and control mice 8 days or 42 days after MI (Fig. 7e).

Recently, a new non-invasive MRI method, longitudinal relaxation time in rotating frame (T_1ρ_) has been introduced to detect granulation and scar tissue formation in myocardial ischemia in mice^26^. Additionally, relaxation times of relaxation along a fictitious field (T_RAFF2_ and T_RAFF4_) are novel rotating frame relaxation time methods for measuring damaged areas of acute and chronic MI scar components with high accuracy and with lower specific absorption rate than T_1ρ_^27^. To analyze the changes in LVW composition after MI, we performed T_1ρ_, T_RAFF2_ and T_RAFF4_ relaxation time cMRI for a subset of mice 7 days after LAD ligation (Fig. 7f,h). All methods were able to differentiate infarcted regions of the LVW from the remote healthy areas. Interestingly, sVEGFR3 mice had significantly increased T_RAFF4_ relaxation times compared to the controls (0.263 s vs. 0.149 s, respectively. P < 0.05) and also a trend towards higher T_RAFF2_ relaxation times (0.0795 s vs. 0.0596 s, respectively) in the infarcted region of LVW (Fig. 7g,h). These findings indicate changes in the composition of infarcted area in sVEGFR3 mice after MI.

## Discussion

We have studied the role of VEGFR3 in cardiac lymphatic vessels and in healing after MI. Confocal microscopy analyses revealed that cardiac lymphatic vessels formed an organized network in the epicardium of the LVW in control mice. In contrast, cardiac lymphatic vessels in sVEGFR3 and Chy mice had more disorganized and dilated morphology. Interestingly, the mortality of the sVEGFR3 mice was significantly higher in the acute phase after MI than their control littermates, emphasizing the importance of VEGFR3 signaling and the function of lymphatic vessels in the healing after MI.

VEGFR3 is the primary lymphangiogenic receptor for growth factors VEGF-C^28^ and VEGF-D^29^. It is expressed in cardiac LECs both during the development and postnatally^9^. In the current study, we used transgenic mice that produce VEGFR3 decoy receptor under the K14 promoter that targets the receptor expression in the keratinocytes of the skin. sVEGFR3 protein is secreted in the blood stream and has shown to cause both metabolic^6,30^ and structural effects^31^ in the lymphatics. Even though VEGFR3 function is not completely lost in sVEGFR3 mice, partial inhibition of VEGF-C and VEGF-D was sufficient for the downregulation of lymphangiogenic signaling leading to modifications in the structure of cardiac lymphatics. Additionally, cardiac lymphatic vessel morphology was significantly altered and lymphatics formed large sheet-like structures in the Chy mice that have an inactivating mutation in the VEGFR3 gene. Unfortunately, the analysis of Chy mice is very challenging due to their poor breeding performance and intolerance for anesthetics. It is likely that the formation of cardiac lymphatic vessels is already altered during embryonic development both in sVEGFR3 and Chy mice. This is supported by the previous findings from other parts of the body showing that many internal organs in the sVEGFR3 newborn mice are devoid of lymphatic vessels^31^ and some newborn Chy mice develop abdominal chylous ascites that is not absorbed due to defective lymphatic network^32^. Unexpectedly, 25% of the sVEGFR3 mice died during the first week after MI whereas control mice survived much better during this acute phase after MI indicating that either the structural changes in cardiac lymphatic system or downregulation of VEGFR3 signaling is detrimental for the healing after MI.

MI causes decreased cardiac lymph flow leading to edema both in humans^33^ and in large animals^34^. Cardiac edema can strongly modulate cardiac function and lead to dangerous ventricular arrhythmias^14^ that are typically the main cause of sudden death after MI^35^. As in previous studies^15,16^, control mice in this study exhibited activated lymphangiogenesis leading to dense lymphatic capillary network. In contrast, sVEGFR3 mice did not respond to VEGF-C upregulation and displayed significantly less lymphatics than control mice. It still remains an open question, whether cardiac lymphatic vessels in sVEGFR3 mice are unable to respond to lymphangiogenic signals after MI or if the pre-existing lymphatics were not functional due to developmental defects. To test this in the future, lymphangiogenesis could be inhibited using conditional transgenic mouse lines or by administering viral vector expressing sVEGFR3 to the myocardium of an adult animal during MI. We assumed that the lower number of cardiac lymphatics would lead to an accumulation of fluids in the heart muscle. T_2_ relaxation times were indeed significantly increased 8 days and 42 days post-MI in both groups but T_2_ relaxation time did not differ between the groups. However, the effects of cardiac edema cannot be totally ruled out as even small increase in the water content of the heart significantly decreases cardiac output^36^. In a recent clinical trial, the activation of both angiogenesis and lymphangiogenesis with adenoviral VEGF-D therapy was shown to be beneficial for myocardial perfusion and might have also improved cardiac fluid balance^17^. Additionally, VEGF-C therapy in a rat model decreased cardiac water content only by 0.8% compared to the controls but was shown to be beneficial for cardiac healing^16^. Therefore, even a slight increase in cardiac fluid accumulation after MI can lead to a higher mortality in sVEGFR3 mice. Additionally, the accumulation of macrophages has been associated with increased vascular leakage and tissue edema^37^ and activation of lymphangiogenesis could help to resolve innate inflammatory reaction^38^. We did not discover differences in the inflammatory cell counts between sVEGFR3 mice and control mice indicating similar responses to inflammatory activation at least in the current study setup. However, VEGFR3 signaling can modulate macrophage polarization^30^, which might modify myocardial healing after MI.

sVEGFR3 mice followed for 8 days displayed intramyocardial hemorrhages indicating microvascular dysfunction and vascular leakage of erythrocytes. Hemorrhages can cause detrimental remodeling and reduced wall motion of the LVW^39^ and they are often discovered in mice with lethal cardiac rupture^40^. Mechanistically, hypoxia after coronary artery occlusion induces the expression of cytokines, growth factors and reactive oxygen species, which can alter the integrity of the microvascular endothelium and lead to increased vascular permeability^41^. The expression of sVEGFR3 led to significantly increased VEGFR2 protein levels in healthy hearts of sVEGFR3 mice compared to controls, which induced the expression of eNOS, one of the main factors regulating vascular permeability^42^. In addition to eNOS, upregulated levels of VEGFR2 can cause increased vascular permeability by decreasing VE-cadherin at endothelial cell junctions without changing vascular structure or density^24^, which might be an additional mechanism also in sVEGFR3 mice. Furthermore, chow-fed sVEGFR3 mice are hypercholesterolemic^6^, which could cause vascular dysfunction and increased vascular permeability in the heart^43^.

Vascular leakage and persisted edema has been shown to modify the development of fibrosis in the heart^20^. Although the expression of ECM proteins, Col1a2, Col3a1 and Postn were at a similar level both in the controls and sVEGFR3 mice, some sVEGFR3 mice had fainter histological staining of collagen I and III after MI. Accordingly, sVEGFR3 mice had a significantly different composition of the infarcted area compared to the controls when the infarcted area was analyzed with novel MRI techniques, T_RAFF2_ and T_RAFF4_ rotating frame relaxation methods. T_RAFFn_ is produced by nested sine amplitude and cosine frequency modulated radio frequency (RF) pulses^44^. The method is based on a fast, sub-adiabatic sweep of effective RF field which produces a fictitious magnetic field. The fictitious field component together with RF pulse induced magnetic field and off-resonance component forms a final effective RF field. Magnetization precesses around this final effective field and relaxes as a function of time^45^. T_RAFF_ has been shown to detect especially slow molecular motions^44^. T_RAFF2_ and T_RAFF3_ relaxation times increased at fibrotic areas in transverse aortic constriction mouse model^45^ and T_RAFF4_ was recently utilized to differentiate white and grey matter and correlate highly with myelin content of the brain^46^ with remarkably lower (approximately 80%) SAR-values of RAFF4 and (approximately 30%) SAR-values of RAFF2 compared to continuous wave T_1ρ_ which is an advantage of RAFFn^44,46,47^. Interestingly, changes in infarcted area of sVEGFR3 mice were not detected with conventional MRI methods, T2 or T1ρ, indicating that T_RAFFn_ could be used to detect more specific modifications in the infarcted area related to chemical exchange of hydrogen between hydroxide groups and free water.

Even though novel imaging methods and transgenic animal models have provided new insights into the function of systemic and tissue-specific lymphatic vessels, the effects of altered cardiac lymphatic function in the healthy hearts and after MI have not been described. Here we show that VEGFR3 has an essential role in cardiac lymphatic vessel morphology and attenuated VEGFR3 signaling exposes mice to higher mortality, hemorrhages and a modified structure of the infarcted area verifying the importance of lymphatic vessel function in the healing after MI. Additionally, we showed that novel MRI techniques provide useful information about the changes in the LVW structure during the myocardial healing and these non-invasive methods could be utilized to determine the scar structures also in the clinical settings.

## Materials and Methods

### Animals

sVEGFR3 and Chy mice in LDLR^−/−^ x ApoB^100/100^ background were bred as previously described^6^. LDLR x ApoB^100/100^ littermates from the breedings with sVEGFR3 mice served as controls. Mice were housed in the Laboratory Animal Center of University of Eastern Finland and they were fed ad libitum with water and normal rodent chow diet. Additionally, to test the effect of high-fat diet on cardiac lymphatic vessel morphology, mice were fed with Western type high-fat diet (TD.88137 Harlan Teklad; 42% of energy from fat, 0.2% cholesterol) for 12 weeks before euthanasia (n = 7/control mice n = 13/sVEGFR3 mice and n = 4/Chy mice). Mice were euthanized with carbon dioxide (CO_2_) and perfused with phosphate-buffered saline (PBS) through the LV. All animal experiments were approved by National Experimental Animal Board of Finland and were carried out in accordance with the Act on the Protection of Animals Used for Scientific or Educational Purposes (497/2013).

### Myocardial infarction (MI)

Approximately 13–17 week old sVEGFR3 mice and control mice (n = 22/group) were used for MI study. Both female and male mice were used in equal numbers. Mice were anesthetized with isoflurane inhalation (Univentor-400, Univentor, Zejtun, Malta) 4% induction followed by 2% maintenance and MI was induced as previously described^48,49^. Briefly, the heart was exposed, pushed out of the thorax with a direct visual control and LAD was ligated at a site ≈5 mm from its origin using a 6-0 silk suture. Mice were sacrificed 8 days after the operation. In addition, some analyses were performed on mice followed for 4 days (n = 6/group) or 42 days (n = 10/control mice and n = 11/sVEGFR3 mice) after MI and a subset of mice used for T_1ρ_ and T_RAFFn_ weighted MRI was followed for 7 days (n = 11/control mice and n = 7/sVEGFR3 mice). In total, 95 mice were used for the MI experiments.

### Cardiac magnetic resonance imaging (cMRI)

MRI was performed before the LAD ligation and during the follow-up (day 3, 8 and 42). All MRI experiments were done using a horizontal 9.4 T magnet controlled by a Bruker console (Bruker GmbH, Ettlingen, Germany). Quadrature volume transceiver with coil inner diameter of 35 mm (Rapid Biomed GmbH, Ettlingen, Germany) was used in MRI experiments. Mice were anesthetized for MRI with 4% isoflurane with oxygen and nitrogen gases by fraction of 1:3 (Piramal Healthcare, Northumberland, UK). The level of isoflurane was decreased to 1–1.5% level during the imaging. Mice body temperature was kept at natural temperature level (37 °C) by circulating warm water in watertubes which were placed under mice. ECG was measured using needle electrodes from fore paws and respiration was controlled by a pneumatic pillow placed under the mouse. Both signals were registered using Model 1025 (Small Animal Instruments Inc., NY, USA) during the experiments. All MRI experiments were gated with ECG and respiration.

Multi-slice cine images were taken to image the whole heart by using gradient echo based fast imaging with steady state precession (FISP) readout sequence. The imaging parameters for cine images were field-of-view = 4 × 4 cm^2^, slice thickness = 1 mm, matrix size = 192 × 192, Time of Echo (TE) = 1.9 ms, Time repetition (TR) = 8.0 ms, scan TR = 99.0 ms, flip angle = 10° and number of frames were 10–11 depending on heart rate, 8–10 slices were imaged depending on the size of the heart. EDV and ESV were analyzed from cine frames with Matlab software using Aedes toolbox. SV was calculated using formula SV = EDV − ESV and EF was calculated using formula EF = (SV/EDV) × 100%.

T_2_ measurements contained Hahn double echo preparation which included an adiabatic half passage (AHP) excitation-pulse (power = 1250 Hz, duration = 3.0 ms), two Hyperbolic Secant 1-pulses (power = 1250 Hz, duration = 4.5 ms) and AHP-backpulse (power = 1250 Hz, duration = 3.0 ms). Between the pulses symmetric delays were used resulting in total TEs of 0.05, 2.3, 4.5, 14.0 ms. Delays in front of T_2_ preparation were 14.01 4.5, 2.3 and 0.05 ms, respectively. Rotating frame preparation module to measure T_RAFFn_ consisted of RAFF2 or RAFF4 pulses (pulses RF power 1250 Hz and 648 Hz, respectively duration 2.26 ms) applied in pulse trains of lengths 0, 9.1, 18.1 and 36.2 ms. In front of the RAFFn pulse trains was a delay with durations of 36.2, 27.15, 18.1 and 0 ms, respectively, to adjust imaging to occur at the same cardiac phase for weightings with different durations. T_1ρ_ preparation was done using a rotating frame preparation module which consisted AHP pulse (power = 625 Hz, duration = 2.0 ms), continuous wave spin-lock-pulse with time-to-spin-lock TSL = 0.4, 9.4, 27.4 and 45.4 ms and AHP-backpulse^26^. Before T_1ρ_ preparation a delay (45.4, 27.4, 9.4 and 0 ms, respectively to TSL) was added. FISP-readout sequence for a single slice was used in all relaxation time measurements with parameters: field-of-view = 4 × 4 cm^2^, slice thickness = 1 mm, matrix size = 256 × 256, flip angle = 90°, TE = 1.9 ms, TR = 14.9 ms, scan was dependent both on the heart rate and the respiratory rate. Minimum delay between weighting pulses was 1460 ms. T_1ρ_ and T_2_ relaxation time maps were fitted by using a linear function. T_RAFF2_ and T_RAFF4_ were fitted by using a single mono-exponential decay function without taking into account the steady state formation.

### Echocardiography and electrocardiography (ECG)

Transthoracic echocardiography was performed on healthy mice and before the sacrification (day 7 or day 35). Mice were anesthetized with isoflurane and imaged using a high-frequency, high-resolution imaging system for small animals (Vevo 2100, VisualSonics, Toronto, Canada) equipped with a transducer probe operating at 18–38 MHz (MS-400, VisualSonics). In addition, surface electrocardiography (ECG) signal was acquired during echocardiography. The paws of the mice were attached to the electrode pads of the heated platform (36–37 °C). ECG data were exported from Vevo software (VisualSonics) and analyzed with rodent ECG imaging software (Kubios HRV 2.0, Kuopio, Finland) as previously^18^.

### Real-time Quantitative PCR (RT-qPCR)

The proximal part of the heart was snap-frozen in liquid nitrogen for molecular biology analyzes. RNA was extracted with RNeasy Mini Kit (Qiagen, Hilden, Germany) and RNase treatment was performed with Turbo DNA-Free^TM^ kit (ThermoFisher Scientific, Waltham, MA, USA) according to the manufacturer’s protocols. RNA was transcribed into cDNA with RevertAid Reverse Transcriptase (ThermoFisher Scientific). Taqman assays (ThermoFisher Scientific) as well as PrimeTime qPCRs (IDT, Coralville, IA, USA) were used to analyze the expression of genes related to lymphangiogenesis and fibrosis.

### Histology and immunohistochemistry

For immunohistology, tissue samples were fixated in 4% paraformaldehyde-PBS overnight. After fixation, approximately 1 mm thick piece of the anterior side of the heart was stored in PBS for confocal microscopy. Samples for histological stainings were processed to paraffin and cut as 4 µm sections.

For visualization of lymphatic vessels, tissues were incubated with a LYVE1 antibody (1:1000, Reliatech, Wolfenbuttel, Germany) with 5% goat serum in PBS containing 0.3% Triton-x100 and 0.2% BSA overnight following washing steps and incubation with a Goat anti-rabbit Alexa 594 (1:500, ThermoFisher Scientific) secondary antibody overnight. For further visualization of LECs, tissues were first incubated with PROX1 antibody (1:50, R&D Systems, Minneapolis, MN, USA) using Donkey anti-goat Alexa 594 (1:500,ThermoFisher Scientific) as a secondary antibody and then with LYVE1 antibody using Chicken anti-rabbit Alexa 488 (1:500,ThermoFisher Scientific) as a secondary antibody. After washes with PBS, tissues were imaged with confocal microscope (Zeiss LSM700, Carl Zeiss, Oberkochen, Germany) or fluorescent microscope (Nikon Eclipse Ni, Nikon, Tokyo, Japan). Lymphatic vessels area in ROI was quantified with ImageJ software.

Infarct sizes and hemorrhages were analyzed from Hematoxylin-Eosin stainings 4, 8 and 42 days post-MI at the level of papillary muscles (3–4 tissue sections/mouse). To analyze post-MI fibrosis, tissue sections were stained with Masson’s trichrome (Sigma-Aldrich, St Louis, MO, USA) and Picro Sirius red (Abcam, Cambridge, UK) according to the manufacturer’s protocols. Immunohistochemical stainings were performed with antibodies recognizing lymphatic vessels (LYVE1, 1:1000), macrophages (F4-80, 1:500, Biorad, Hercules, CA, USA), lymphocytes (CD45, 1:50, BD Biosciences, San Jose, CA, USA) and α-sma (anti-alpha smooth muscle actin-Cy3, 1:200, Sigma-Aldrich). Blood capillaries were stained with biotinylated lectin (Biotinylated Griffonia (Bandeiraea) Simplicifolia Lectin I, 1:100, Vector Laboratories). To improve unmasking of the antigen and enhance the intensity of the staining, antigen retrieval with boiling in citrate buffer was used for LYVE1 and F4-80 antibodies and BD Retrievagen A solution (BD Biosciences) for CD45 antibody. To visualize the binding of the antibody, tissue sections were incubated with biotinylated IgG secondary antibodies (1:500, Vector Laboratories, Burlingame, CA, USA) followed by avidin-biotin-HRP step (Vector Laboratories) and a choromogen DAB (ThermoFisher Scientific). Tissue slides were mounted with Permount (ThermoFisher Scientific) for light imaging or Vectashield mounting medium with DAPI (Vector Laboratories) for fluorescent imaging. Tissue sections were imaged with NIS elements software (AR5.50.00, Nikon) connected to a light microscope (Nikon Eclipse Ni, Nikon). Image analyses were performed with ImageJ software equipped with Fiji image processing package.

### Protein extraction and western blotting

Proteins were extracted using T-PER Tissue Protein Extraction Reagent (Thermo Fisher Scientific) and total protein content was determined using BCA protein assay (Pierce, Thermo Fisher Scientific). 40 µg of protein was separated on Mini-PROTEAN TGX Stain-Free gels (BioRad). Gel was activated by UV for 2.5 min and proteins were transferred to nitrocellulose membrane (BioRad). The membrane was blocked in TBST (Tris-buffered saline with Tween 20) containing 5% nonfat dry milk and incubated with a rabbit anti-mouse VEGFR2 (Cell Signaling) primary antibody and a goat anti-rabbit secondary antibody (Thermo Fisher Scientific). Total protein amount was measured with ChemiDox XRS before enhanced chemiluminescence (ECL) detection. Specific bands were normalized to total protein using the ImageLab (Bio-Rad) software.

### Statistical analyses

Two-tailed unpaired t-test, one-way or two-way ANOVA followed by Bonferroni correction when appropriate. Survival curves were created using Kaplan-Mayer method and survival curves were compared with a log-rank test. Data is presented as mean ± SEM and P < 0.05 was considered significant. Statistical analyses was performed with GraphPad Prism software (GraphPad Software, La Jolla, CA, USA).

## Data Availability

No datasets were generated or analyzed during the current study.
